# *Aspilia africana* (Pers.) C.D. Adams and *Manihot esculenta* Crantz Exhibit Antibacterial Activity against Resistant *Salmonella typhi* Strains

**DOI:** 10.1155/2024/6637904

**Published:** 2024-08-26

**Authors:** Richard Agyen, Yaw Duah Boakye, Theresa Appiah Agana, Vivian Etsiapa Boamah, Clement Osei Akoto, Michael Owusu, Francis Adu, Christian Agyare

**Affiliations:** ^1^ Department of Pharmaceutics Kwame Nkrumah University of Science and Technology, Kumasi, Ghana; ^2^ Department of Chemistry Kwame Nkrumah University of Science and Technology, Kumasi, Ghana; ^3^ Department of Medical Diagnostics Kwame Nkrumah University of Science and Technology, Kumasi, Ghana

## Abstract

Typhoid fever, caused by *Salmonella typhi*, has plagued underdeveloped countries for many years. Recently, there has been a surge in *S. typhi* strains identified to be multidrug-resistant in endemic areas. *Aspilia africana* and *Manihot esculenta* have been reported to exhibit activity against *S. typhi*; however, this study aimed to investigate the effect of *A. africana* and *M. esculenta* against resistance strains of *S. typhi.* The leaves of the plants were extracted using distilled water (hot (A_QH_) and cold (A_QC_)), methanol (M_ET_), ethyl acetate, and petroleum ether. The extracts were screened *in vitro* for anti-*Salmonella* effects against fourteen *S. typhi* isolates (five multidrug-resistant (MDRST), five ciprofloxacin-resistant (CRST), three nalidixic acid-resistant (NARST), and one sensitive isolate (SS)) using agar well diffusion and microbroth dilution methods. Phytochemical screening showed the presence of tannins, saponins, glycosides, and flavonoids in all polar solvent extracts. Alkaloids were found in all extracts, while triterpenoids were present in all except the aqueous extracts. The A_QC_ of *A*. *africana* had the best inhibitory effect on the MDRST and NARST with diameter zones of inhibitions (DZOIs) of 40.0 ± 2.08 mm and 34.0 ± 3.22 mm, respectively. Methanol extract of *A*. *africana* had the best inhibitory effect on CRST and SS with DZOIs of 34.0 ± 2.08 and 43.0 ± 3.06 mm, respectively. The A_QC_ and A_QH_ of *A*. *africana* and A_QH_ of *M*. *esculenta* produced the best MICs and MBCs of 2.5 and 5.0 mg/mL against the MDRST. There was no significant difference in ZOIs of the different solvent extracts against test organisms at *p* < 0.05. Gas chromatography-mass spectrometry analysis of the extracts showed compounds such as n-hexadecanoic acid, 9,12,15-octadecatrienoic acid (9.55%), and 2H-benzo[F]oxireno[2,3-E]benzofuran-8(9H)-one in the *A. Africana* extracts and D-mannose, 3-nitrophenyl, methanol acetate (ester), and 9-octadecenamide in the *M. esculenta* extracts. The leaves of *M. esculenta* and *A. Africana* are effective against multidrug-resistant *Salmonella* isolates.

## 1. Introduction

Typhoid fever is a disease that affects people all over the world, although it is most common in places with inadequate sanitation. According to experts, it is one of the most underreported diseases in the developing world. For instance, in Ghana, reported cases of typhoid fever show a disturbing trend and a major public health concern. Typhoid fever was one of the top twenty causes of outpatient morbidity and 1.2, 1.7, and 1.3% of hospital admissions in 2017, 2016, and 2015, with 365,148, 384,704, and 337,120 cases, respectively [1], in Ghana.

In recent years, there has been a surge in the number of *S. typhi* resistance strains, especially in endemic areas [2]. The development of multidrug-resistant (MDR) *S. typhi* strains has worsened the condition leading to high morbidity and mortality rates in most developing countries due to the increased ineffectiveness of antibiotics indicated for treating *S. typhi* infection [3]. Hence, there is a need to search for and develop new drugs from natural products to salvage the menace. Natural products such as medicinal plants are still widely used in most developing countries as a reliable source of traditional medicine due to the belief that they are safe and efficacious [4]. However, most of these plants have not been scientifically evaluated to give credence to their folkloric use in Ghana.

*Aspilia africana* is an indigenous plant used by traditional medicine practitioners in Africa to cure certain illnesses [5]. It is known as “nfofo” in Akan, “organgila” in Ibo, “tazalian” in Hausa, “yungung” in Yoruba, and “Makayi” in Luganda (Uganda) [6]. It has many traditional uses for its leaves and flowers but the most famous is its use to halt bleeding and accelerate healing of wounds. As a result of its capacity to halt bleeding from recent wounds, it is known as the “haemorrhage plant” [3]. It contains biologically active substances that are antiviral, fungicidal, and antibacterial [3]. Flavonoids, saponins, tannins, alkaloids, D-, *α*-pinene, carene, phytol, linolenic acid, *β*-caryophyllene, and germacrene have been reported to confer anti-inflammatory, antimicrobial, and antioxidant activities on *A. africana* [7]. Some studies have been carried out on the antimicrobial activity of *A. africana*. For instance, the antibacterial activity of *A. africana* against some clinical isolates of *Salmonella typhi* has been reported [8]. Anibijuwon et al. [9] and Halimat et al. [10] also reported that *A. africana* exhibits activity against other pathogenic microorganisms.

*Manihot esculenta*, commonly called cassava, is a tuberous woody shrub of the *Euphorbiaceae* (spurge) family. Cassava is a perennial woody shrub native to South America and also cultivated in tropical and subtropical zones of the world. It is one of the most vital industrial food crops in the world [3]. Cassava tube is utilized as a staple meal by the general public, but the leaves are used to combat and treat a variety of ailments, including rheumatism, gout, diarrhoea, fever, headache, night blindness, intestinal worms, and *beriberi* [11]. In addition to treating skin rashes and fevers, the leaves are also used to cure ringworm, tumours, conjunctivitis, and abscesses. Fresh rhizomes and leaf sap latex are used for eye ulcers and rheumatism, respectively [12, 13]. Studies indicate that cassava leaf paste contains phytochemicals such as flavonoids, tannins, saponins, sitosterol, and stigmasterol, which contribute to its antioxidant, antimicrobial, and anti-inflammatory properties [14]. The ethanol extract of *M. esculenta* has been reported to exhibit activity against *Escherichia coli and Salmonella* spp. on contaminated meat [15]. Mustarichie et al. [16] reported on the activity of *M. esculenta* leaves against clinical isolates of *Staphylococcus epidermidis* and *Propionibacterium acnes*. The antibacterial activity of cassava *M. esculenta* leaf extract against *Escherichia coli* has been reported [17].

Plants have been used for the management of diverse kinds of man's health conditions all over the world from time immemorial. In Ghana, medicinal plants are widely used to treat typhoid fever in both rural and urban settings [18]. Extracts from the leaves, barks, seeds, and fruits of plants including *A. africana* and *M. esculenta* are used in the preparation of syrups and infusions in traditional medicine for the treatment of ailments such as typhoid fever. Information about the use of these plants in the Ghanaian Traditional practice was complemented with interviews with some local people who always use them to treat typhoid fever [19, 20]. Some of these medicinal plants have been screened for their activity against typed strains of *S. typhi*.However, most of these plants have not been screened for their anti-*Salmonella* activity against resistant strains which are now responsible for most of the typhoid infections reported in hospitals. This study, therefore, screened different solvent extracts of *A. africana* and *M. esculenta* against drug-resistant strains of *S. typhi* and gave credence to the continuous use of these plants in folkloric medicine for treating typhoid fever.

## 2. Materials and Methods

The following is a schematic representation of the methods used in assessing the anti-*Salmonella* activity of leaves of *Aspilia africana* and *Manihot esculenta* against selected drug-resistant strains of *S. typhi*.


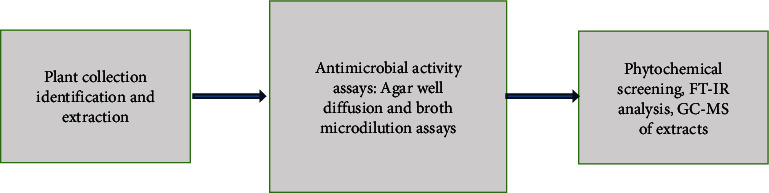


### 2.1. Plant Collection

Leaves of *Aspilia africana* and *Manihot esculenta* were collected in March 2020 at Dunkwa-on-Offin (5°58′11.3232″N and 1°46′59.1492″W) in the central region of Ghana. They were identified at the Department of Herbal MedicineKwame Nkrumah University of Science and Technology, Kumasi. Leaves were given voucher specimen numbers KNUST/HMI/2020/L016 and KNUST/HMI/2020/L017, respectively.

### 2.2. Preparation of Extracts

The solvents used were selected based on increasing polarity (petroleum ether, ethyl acetate, and methanol). Aqueous extracts (both hot and cold) that are often used traditionally in the management of diseases were also prepared.

The fresh leaves of *A. africana* and *M. esculenta* were washed under running tap water to remove debris and made to drain before air drying under shade for two weeks. The dried plant materials were ground mechanically with a mortar and pestle into smaller particles and ground into fine particles using a blender. Aqueous (hot and cold), methanol, ethyl acetate, and petroleum ether extracts were prepared by suspending 200 g each of finely ground leaves in 1000 mL of the respective solvents. The solutions in the flasks were corked with cotton wools, covered in aluminium foils, and left at room temperature for 3 days with intermittent shaking. The resulting solutions were filtered using Whatman no. 1 filter paper and the filtrates were kept in a rotary evaporator at 50°C for complete evaporation of the solvents. The extracts were then stored in a refrigerator at 4°C. With the hot water extraction, 200 g of the ground leaves were suspended in 1 litre of sterile water and heated on a burner at 100°C for 45 minutes. The resulting solution was allowed to cool and filtered using Whatman no. 1 filter paper and the filtrate was lyophilized.

### 2.3. Test Microorganisms

Microorganisms used for this study were obtained from the Kumasi Centre for Collaborative Research in Tropical Medicine (KCCR) in well-labelled sterile nutrient broths. They included five multidrug-resistant (MDRST) (G10163, G10209, G10325, G10573, and G10194), three nalidixic acid-resistant (NARST) (G20014, G10037, and G20157), five ciprofloxacin-resistant (CRST) (G10628, G20247, G11082, G10692, and G10713), and one sensitive strain (SS) *S. typhi*. They were stored in the refrigerator at 4°C. The *S. typhi* strains were streaked on bismuth sulphite agar (BSA) to determine their characteristic appearance (observance of rabbit eye colonies with black metallic sheen). Gram stain and microscopy were used to ascertain whether the organisms were Gram-negative or positive bacteria. Further confirmatory tests were carried out on well-separated colonies grown on BSA using the sulfide indole motility (SIM), catalase, citrate utilization, methyl red-Voges Proskauer (MR-VP), and triple sugar iron agar (TSI) tests. Pure isolates of each organism were inoculated into 10 mL of sterile water and compared to 0.5 M McFarland standard to standardize the approximate number of organisms by comparing their turbidity. Before each experiment, a 24-hour broth culture of the *S. typhi* (1 ∗ 10^6^ CFU/mL) was prepared.

### 2.4. Determination of the Susceptibility of Organisms to Extracts

The agar well diffusion method described by Boakye et al. [21] was used to determine the susceptibility of the test organisms to the extracts. In this method, the crude extracts were prepared in various concentrations of 100, 30, and 10 mg/mL. Ciprofloxacin (10 *µ*g/mL) was used as a positive control. The inoculum size of 1.0 × 10^6^ CFU/mL of the test organisms was used in all antibacterial determinations. Sterile cotton swabs were used to evenly streak 1 mL of each organism onto their respective Muller–Hinton agar plates. An 8 mm cork-borer was flamed and used to aseptically create 4 wells equidistant from each other in each agar plate. A volume of 150 *µ*L of each concentration of the various extracts and ciprofloxacin were aseptically filled into their, respectively, labelled wells using a micropipette. The plates were left at room temperature for a diffusion time of 45 minutes before incubating them at 37°C for 24 hours. The plates were observed after the incubation period for clear zones and these were measured with a ruler as the zones of growth inhibition.

### 2.5. Determination of the Minimum Inhibitory Concentration (MIC) and the Minimum Bactericidal Concentration (MBC) of the Extracts

The minimum inhibitory concentration (MIC) and minimum bactericidal concentration (MBC) of the extracts were determined using the microbroth dilution method by Boakye et al. [21]. A 96-well microtitre plate was used, and 100 *µ*L of double-strength nutrient broth was added to each well. A stock of 200 mg/mL of each extract was prepared and calculated volumes of the stock solution needed to produce the required concentrations (80, 40, 20, 10, 5, and 2.5 mg/mL) in each well were added. Calculated volumes of sterile water and 20 *µ*L (1 ∗ 10^6^ CFU/mL) of the inoculum were added to the appropriate wells to produce the needed concentrations. Ciprofloxacin was used as positive control and prepared in 10, 3, and 1 *µ*g/mL. The plates were incubated at 37°C for 24 hours. After incubation, 20 *µ*L of 1.25 mg/mL MTT was added to each well and incubated at 37°C for 30 minutes to check for growth. A purple colour change indicated the growth of organisms and pale to yellow showed the absence or no growth of organisms. The least concentration that inhibited growth (exhibited no change of colour from pale yellow to purple) was recorded as the MIC. For the MBC determination, a loopful of cultures were taken from the wells that showed inhibitory activity and subcultured in sterile nutrient broths and incubated at 37°C for 48 hours followed by the addition of 1.25 mg/mL MTT to each well. The wells which contained the least concentrations that exhibited no growth after the incubation period were considered as the MBC. The experiments were carried out in triplicate.

### 2.6. Determination of Phytochemical Constituents

The plant extracts were screened for the presence of tannins, saponins, flavonoids, glycosides, alkaloids, triterpenoids, and steroids according to the method described by Evans [22] and Gul et al. [23].

### 2.7. Fourier-Transformed Infrared Spectrometry of the Extracts

This was performed to determine the functional groups for the identification of compounds in the plants. One milligram of each extract was mounted directly on the KBr disc of the PerkinElmer 200 UATR (FTIR) spectrophotometer. It was then scanned through IR regions between the ranges of 400 and 4000 cm^−1^ at a resolution of 4 cm^−1^ [24].

### 2.8. Gas Chromatography-Mass Spectrometry Analysis of the Extracts

An amount of 1 mg of each extract was analyzed by GC-MS. The analysis was carried out using a PerkinElmer Clarus 580 and (Clarus SQ 8 S) GC/MS with an Elite 5 MS column that has a dimension of 30 × 0.25 m ID × 0.25 m DF and is 5% diphenyl/95% dimethylpolysiloxane. The injector and ion source were kept at respective temperatures of 250°C and 150°C. With an ionization energy of 70 electronvolts (Ev) and helium gas that was 99.99% pure acting as the carrier gas, the GC-MS detection was run in electron impact mode with a constant flow rate of 1 mL/min. The bioactive fractions' mass spectra were read at 70 Ev, scanned in 0.5 seconds, and then fragmented by their mass-to-charge ratio from 45 to 450 Da. A TurboMass detector was used to find the separated ions, and TurboMass version 6.1.0 software was used to record the chromatogram by amplifying the signals from the spectra. To initialize the system, the solvent was started at 0 minutes and delayed for 2 minutes before being run for 47 minutes on the GC-MS. The determined bioactive fractions' mass spectra were analyzed, and they were contrasted with the GC-MS spectrum database maintained by the National Institute of Standards and Technology (NIST).

## 3. Results

### 3.1. Percentage Yields of Plant Extracts

The hot aqueous (A_QH_) extract of *A. africana* gave the highest yield (31.02%), followed by cold aqueous (25.42%), methanol (20.95%), and ethyl acetate extracts (13.68%). Petroleum ether extract had the least yield (10.25%). The percentage yields of 32.28, 35.63, 24.78, 14.64, and 12.30% were obtained for the aqueous hot, aqueous cold, methanol, ethyl acetate extract, and petroleum ether extracts of *M. esculenta*, respectively. All solvent extracts of *M. esculenta* had higher yields than *A. africana* (Table 1).

### 3.2. Preliminary Phytochemical Screening of Plant Extracts

The tests were carried out to give an idea about the secondary metabolites present in the plants. Phytochemical screening showed the presence of saponins, glycosides, tannins, and flavonoids in all the polar solvent extracts but not the nonpolar. Alkaloids were present in all extracts, while triterpenoids were present in all except the aqueous extracts. Sterols were absent in all the extracts (Table 2).

### 3.3. Fourier-Transform Infrared Spectrometry (FTIR) Analysis

The FTIR analysis of the various plant extracts revealed peaks with their respective functional groups. *A*. *africana* had peaks ranging between 970.37 and 3388.46 cm^−1^ with S=O bending, C-N stretching, O-H bending, C=C bending, Csp3-H stretching, O-H stretch, C-O stretching, C-H stretching, N-H stretching, and C-H bending as predictable functional peaks present (Table 3). *M*. *esculenta* had peaks ranging between 972.89 and 3234.28 cm^−1^ with CO-O-CO/S=O stretch, S=O/C-O/C-N stretch, C=C stretch, Csp3-H stretch, O-H stretch, C-O stretch, O-H bending, C=O stretch, C=C bending, and C-H bending as predictable functional peaks present (Table 4).

### 3.4. Antimicrobial Activity of Various Solvent Extracts of *A. africana* and *M. esculenta* against Multidrug-Resistant *S. typhi* (MDRST)

**Figure 1:**
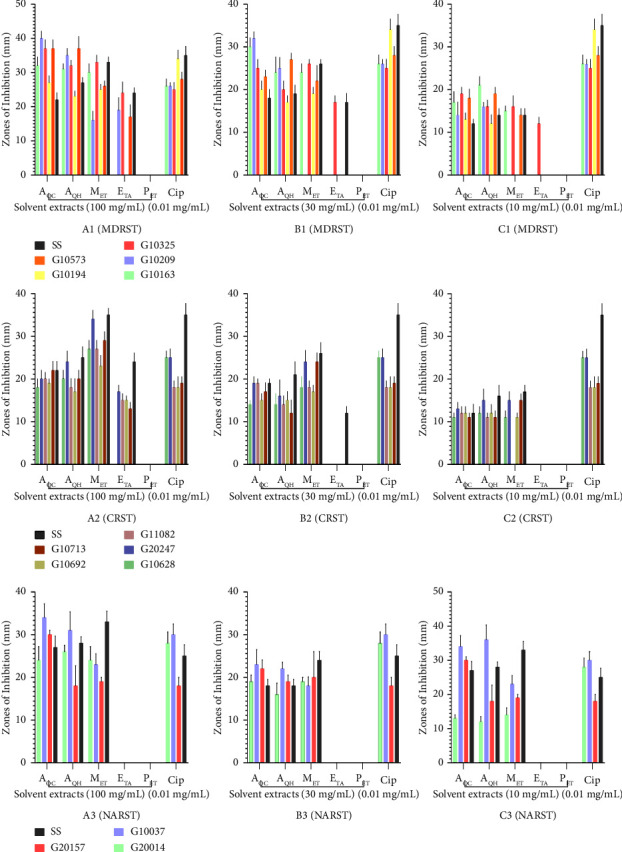
Zones of inhibition of different extracts of *A. africana* against multidrug-resistant, ciprofloxacin-resistant, and nalidixic acid-resistant *S*. *typhi* (MDRST, CRST, and NARST, respectively). A1, B1, and C1; A2, B2, and C2; and A3, B3, and C3 are zones of inhibition of solvent extracts at 100, 30, and 10 mg/mL, respectively, against the various strains of *S. typhi*. A1, B1, and C1 = MDRST; A2, B2, and C2 = CRST; A3, B3, and C3 = NARST strains. A_QC_: cold aqueous extract; A_QH_: hot aqueous extract; M_ET_: methanol extract; E_TA_: ethyl acetate extract; P_ET_: petroleum ether extract; Cip: ciprofloxacin; SS: sensitive strain; *n* = 3; values are expressed as mean ± SEM. There was no significant difference in the zones of inhibition of the different solvent extracts at *p* < 0.05 (one-way ANOVA test).

The highest concentration (100 mg/mL) of methanol and cold and hot aqueous extracts of *A. Africana* showed zone of inhibition (ZOI) ranges of 16 ± 2.65–33 ± 2.08, 27 ± 2.00–40 ± 2.08, and 23 ± 1.53–37 ± 3.51 mm against the MDRST strains and 33 ± 1.52, 22 ± 2.00, and 24 ± 1.53 mm against the sensitive strain (SS), respectively. The petroleum ether extract of *A. Africana* had no activity against any of the MDRST strains at all test concentrations (Figure 1, A1).

**Figure 2:**
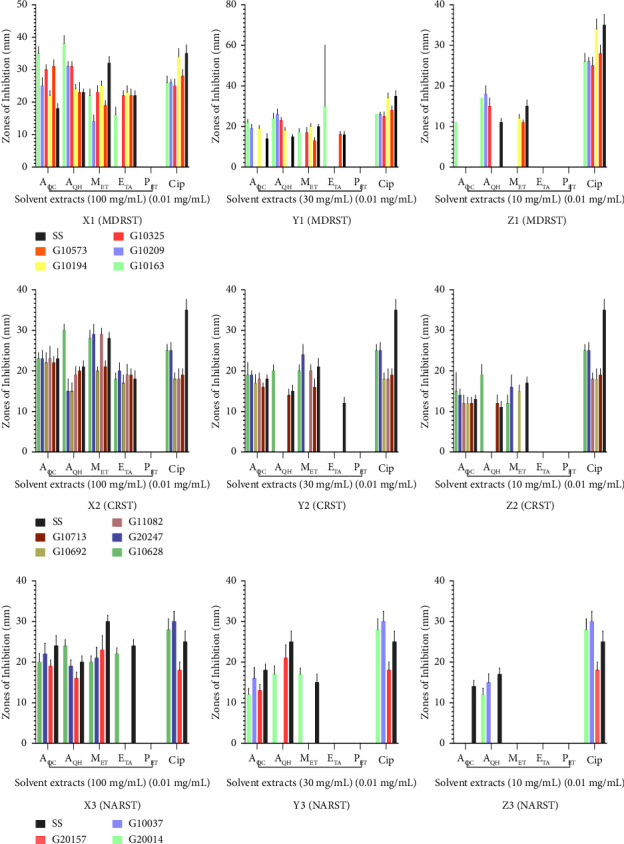
Zones of inhibition of different solvent extracts of *M. esculenta* against multidrug-resistant, ciprofloxacin-resistant, and nalidixic-resistant S. *typhi* (MDRST, CRST, and NARST, respectively). X1, X2, and X3; Y1, Y2, and Y3; and Z1, Z2, and Z3 are zones of inhibition of solvent extracts at 100, 30, and 10 mg/mL, respectively, against the various strains of S. *typhi*. X1, Y1, and Z1 = MDRST; X2, Y2, and Z2 = CRST; X3, Y3, and Z3 = NARST strains. A_QC_: cold aqueous extract; A_QH_: hot aqueous extract; M_ET_: methanol extract; E_TA_: ethyl acetate extract; P_ET_: petroleum ether extract; C_ip_: ciprofloxacin; SS: sensitive strain; *n* = 3; values are expressed as mean ± SEM. There was no significant difference in the zones of inhibition of the different solvent extracts at *p* < 0.05 (one-way ANOVA test).

The highest concentration (100 mg/mL) of methanol and cold and hot aqueous extracts of *M. esculenta* at 100 mg/mL showed ZOI ranges of 19 ± 1.53–25 ± 1.53, 22 ± 1.53–35 ± 2.08, and 23 ± 3.01–38 ± 2.52 against the MDRST strains and 32 ± 2.08, 18 ± 1.53, and 23 ± 1.00 against the SS, respectively. The petroleum ether extract of *M. esculenta* had no activity against any of the MDRST strains at all test concentrations. Ciprofloxacin (0.01 mg/mL), the control antibiotic, showed a ZOI range of 26 ± 1.00–34 ± 2.52 and 35 ± 2.65 against the MDRST strains and SS, respectively (Figure 2, X1).

### 3.5. Antimicrobial Activity of Various Solvent Extracts of *A. africana* and *M. esculenta* against Ciprofloxacin-Resistant *S. typhi* (CRST)

The highest concentration (100 mg/mL) of methanol, cold aqueous, hot aqueous, and ethyl acetate extracts of *A. Africana* showed zones of inhibition (ZOI) ranges of 23 ± 2.52–34 ± 2.08, 18 ± 2.00–22 ± 2.08, 17 ± 3.06–24 ± 2.52, and 13 ± 1.53–17 ± 1.53 against the CRST strains and 35 ± 1.53, 22 ± 2.08, 25 ± 2.52, and 24 ± 2.08 mm against the sensitive strain (SS), respectively.

The petroleum ether extract of *A. africana* showed no activity against any of the CRST strains at all test concentrations (Figure 1, A2).

The highest concentration (100 mg/mL) of methanol, cold aqueous, hot aqueous, and ethyl acetate extracts of *M. esculenta* showed ZOI ranges of 20 ± 1.00–29 ± 2.52, 22 ± 1.53–23 ± 1.53, 15 ± 2.08–30 ± 1.5 and 17 ± 2.00–19 ± 1.53 mm against the CRST strains, and 28 ± 1.53, 23 ± 2.52, 21 ± 1.53, and 18 ± 2.08 mm against the SS, respectively. The petroleum ether extract of *M. esculenta* showed no activity against any of the CRST strains at all test concentrations. Ciprofloxacin (0.01 mg/mL), the control antibiotic, showed ZOI ranges of 18 ± 1.53–25 ± 1.53 and 35 ± 2.65 against the CRST strains and the SS, respectively (Figure 2, X2).

### 3.6. Antimicrobial Activity of Various Solvent Extracts of *A. africana* and *M. esculenta* against Nalidixic Acid-Resistant *S. typhi* (NARST)

The highest concentration (100 mg/mL) of methanol and cold and hot aqueous extracts of *A. africana* showed zone of inhibition (ZOI) ranges of 19 ± 1.00–24 ± 3.22, 24 ± 3.22–34 ± 3.22, and 18 ± 4.73–31 ± 4.36 mm against the NARST strains and 33 ± 2.52, 27 ± 2.65, and 28 ± 1.53 mm against the sensitive strain (SS), respectively. The ethyl acetate and petroleum ether extract of *A. africana* showed no activity against any of the NARST strains at all test concentrations (Figure 1, A3).

The highest concentration (100 mg/mL) of methanol and cold and hot aqueous extracts of *M. esculenta* at 100 mg/mL showed ZOI ranges of 20 ± 1.53–23 ± 3.51, 19 ± 1.53–22 ± 2.65, and 16 ± 1.53–24 ± 1.53 mm against the NARST strains and 30 ± 1.53, 24 ± 2.52, and 20 ± 1.53 against the SS, respectively. The petroleum ether extract of *M. esculenta* showed no activity against any of the NARST strains at all test concentrations. Ciprofloxacin (0.01 mg/mL), the control antibiotic, showed a ZOI range of zones of 18 ± 2.00–30 ± 2.52 and 35 ± 2.65 against the NARST strains and the SS, respectively (Figure 2, X3).

### 3.7. MIC of Extracts of *A. africana* and *M. esculenta* against Test Organisms

The hot aqueous extract of *A. africana* showed the best MIC (2.5 mg/mL) against the MDRST strains, G10163, G10209, and G10325. The cold aqueous extract of *A. africana* also had the best MIC (2.5 mg/mL) against the MDRST strain, G10209. The methanol and hot aqueous extracts of *A. africana* showed the best MICs of 5 mg/mL each against the sensitive strain (SS).

The cold aqueous extract of *A. africana* showed the best MIC of 5 mg/mL each against the CRST strains, G10628, G20247, and G11082. The hot aqueous extract of *A. africana* also had the best MIC (5 mg/mL) against the CRST strains, G10628 and G20247. The methanol and hot aqueous extracts of *A. africana* showed the best MICs of 5 mg/mL each against the CRST strain, G10713, and the sensitive strain (SS).

The cold aqueous extract of *A. africana* showed the best MIC of 5 mg/mL each against the NARST strains, G10037 and G20157. The methanol and hot aqueous extracts of *A. africana* also had the best MIC (5 mg/mL) against the NARST strain, G20014, and the sensitive strain (SS) (Table 5).

The cold aqueous extract of *M. esculenta* showed the best MIC (2.5 mg/mL) against the MDRST strains, G10163 and G10209. The hot aqueous extract of *M. esculenta* showed the best MIC of 5 mg/mL against the sensitive strain (SS).

The methanol and cold aqueous extract of *M. esculenta* showed the best MICs (5 mg/mL each) against the CRST strain, G20247. Only the methanol extract of *M. esculenta* had the best activity against the sensitive strain (SS) with a MIC of 5 mg/mL.

Only the methanol extract of *M. esculenta* had the best MIC (5 mg/mL) against the NARST strain, G20157 (Table 5).

### 3.8. MBC of Extracts of *A. africana* and *M. esculenta* against Test Organisms

The hot aqueous extract of *A. africana* showed the best MBC (5 mg/mL each) against the MDRST strains, G10163, G10209, and G10325. The cold aqueous extract of *A. africana* also had the best MBC (5 mg/mL) against the G10209 strain.

The cold aqueous extract of *A. africana* showed the best MBC of 10 mg/mL each against the CRST strains, G10628, G20247, and G11082. The hot aqueous extract of *A. africana* also had the best MBC (10 mg/mL each) against the CRST strains, G10628, G20247, and G10713. Both methanol and hot aqueous extracts of *A. africana* showed the best MBCs of 10 mg/mL each against the sensitive strain (SS).

The cold aqueous extract of *A. africana* showed the best MBC (10 mg/mL each) against the NARST strains, G10037 and G20157. The methanol and hot aqueous extracts of *A. africana* had the best MBC (10 mg/mL each) against the CRST strain, G20014, and the sensitive strain (SS) (Table 6).

The hot aqueous extract of *M. esculenta* showed the best MBC (5 mg/mL each) against the MDRST strains, G10163 and G10209.

The methanol and cold aqueous extract of *M. esculenta* showed the best MBC of 10 mg/mL each against the CRST strain, G20247. Only the methanol extract of *M. esculenta* showed the best MBC of 10 mg/mL against the sensitive strain (SS).

Only the cold aqueous extract of *M. esculenta* had the best MBC (10 mg/mL) against the NARST strain, G20157 (Table 6).

### 3.9. Gas Chromatography-Mass Spectrometry Analysis (GC-MS) of *A. africana* and *M. esculenta*

GC-MS was used to separate, quantify, and analyze volatile chemicals that may be responsible for the therapeutic effect of the plant extracts.

GC-MS analysis of the methanol (M_ET_) extract of *A. africana* showed 13 compounds with n-hexadecanoic acid in larger quantities (19.29%), followed by 9,12,15-octadecatrienoic acid (9.55%). The cold aqueous (A_QC_) extract showed 5 spots with phosphorothioic acid in larger quantities (29.71%), followed by 18-pentatriacontanone (1.71%). The hot aqueous (A_QH_) extract showed two compounds (cis-11-eicosenamide (5.05%) and hexadecanoic acid (2.90%)). Ethyl acetate extract showed 16 compounds with hexasiloxane 1,1,3,3,5,5,7,7,9,9,11,11-dodecamethyl in larger quantities (22.11%), followed by stigmasterol (18.70%) (Table 7).

The GC-MS analysis of the methanol (M_ET_) extract of *M. esculenta* showed 3 compounds with 9,12, 15-octadecatrienoic acid (Z, Z, Z) in larger quantities (12.78%), followed by n-hexadecanoic acid (8.69%). The cold aqueous (A_QC_) extract showed nine spots with D-mannose in larger quantities (11.84%), followed by (3-nitrophenyl) methanol acetate (ester) (6.51%). The hot aqueous (A_QH_) extract showed nine compounds with octadecanoic acid (37.59%) and 9-octadecenamide (9.59%) in larger quantities. The ethyl acetate (E_TA_) extract showed 12 compounds with octasiloxane 1,1,3,3,5,5,7,7,9,9,11,11,13,13,15,15-hexadecamethyl in larger quantities (42.46%), followed by 6a,14a-methanopicene, perhydro-1,2,4,6b,9,9,12a-heptamethyl-10-hydroxy (8.24%) (Table 8).

## 4. Discussion

Medicinal plants have been used in the past and are still utilized for their therapeutic benefits. *Aspilia africana* and *Manihot esculenta* are plants that are traditionally used in Ghana for the management and treatment of typhoid infections. This study sought to assess the effects of extracts of *A. africana* and *M. esculenta* on resistant strains of *Salmonella typhi.* The solvents used in the extraction process were aqueous (cold and hot), methanol, ethyl acetate, and petroleum ether. These solvents were used based on their eluotropic series to extract a wide range of bioactive compounds. Many medicinal plant parts are boiled locally before they are used to treat various ailments, while others are left in cold water and alcohol, respectively, for days before they are taken. Research has shown that a combination of ethanol and water gives a better extraction yield of phytochemicals [25]. The extraction of the plants studied showed that the solvents used were able to extract most phytoconstituents but with varying amounts.

All solvent extracts of *A. africana* and *M*. *esculenta* had different yields. This confirms the report that differences in the polarity of solvents affect the yield of extracts [26]. In a study conducted by Oko and Agiang [27], the percentage yield after extracting the leaves of *A. africana* with water at room temperature was calculated to be 10.2%, which is lower than the yield obtained in this study (25.42%). This could be due to differences in methods of preparation and geographical locations of the plant samples [28].

On an individual plant basis, the hot aqueous extracts of both *A. africana* and *M*. *esculenta* had the highest percentage yields, which may be due to the application of heat that increased the release of the various plant phytoconstituents into the water used for the extraction. From the percentage yield results, it could be inferred that the hot water was found to be the best extraction solvent for both *A. africana* and *M*. *esculenta*, followed by aqueous cold, methanol, and ethyl acetate.

The antibacterial activity of the various plant extracts was determined against all fourteen organisms using the cup-plate agar diffusion method. The various extracts of the leaf of *A*. *africana* and *M*. *esculenta* had inhibitory effects against the multidrug-resistant, ciprofloxacin-resistant, nalidixic acid-resistant, and the sensitive strain *S. typhi* (MDRST, CRST, NARST and SS, respectively) at the test concentrations (100, 30, and 10 mg/mL). The highest respective concentrations of almost all extracts of *A. africana* and *M*. *esculenta* gave the highest zones of inhibition. This finding is in agreement with a study by Oluduro and Omoboye [29], which reported that the antibacterial activities of most plant extracts are concentration-dependent, as the zone of growth inhibition increases with increasing concentration of the extracts. Kambar et al. [30] also reported that the efficacy of most plant extracts is concentration-dependent.

There were differences in the zones of inhibitions (ZOIs) recorded for the polar solvent extracts of both *A. africana* and *M*. *esculenta*. For instance, the cold aqueous extract of *A. africana* at 100 mg/mL had the highest ZOI (40 ± 2.08 mm), followed by the hot aqueous extract (37 ± 3.51 mm) and the methanol extract (33 ± 2.08 mm) against an MDRST strain (Figure 1, A1). The hot aqueous extract of *M. esculenta* had the best ZOIs of 38 ± 2.52 mm, followed by the cold aqueous (35 ± 2.08 mm) and the methanol extract (25 ± 1.53 mm) against the MDRST strain (Figure 2, X1). The best ZOI against a CRST strain was exhibited by the methanol extract (34 ± 2.08 mm) of *A. africana,* at 100 mg/mL, followed by the hot aqueous extract (24 ± 2.52 mm) and the cold aqueous extract (22 ± 2.08 mm) (Figure 1, A2). The hot aqueous extract of *M. esculenta* had the best ZOIs of 30 ± 1.53 mm, followed by the methanol (29 ± 1.53 mm) and cold aqueous extract (23 ± 1.53 mm) against a CRST strain (Figure 2, X2).

The best ZOI against a NARST strain was exhibited by the cold aqueous extract (34 ± 3.22 mm) of *A. africana,* at 100 mg/mL, followed by the methanol (33 ± 2.52 mm) and the hot aqueous extract (28 ± 1.53 mm) (Figure 1, A3). The hot aqueous extract of *M. esculenta* had the best ZOIs of 24 ± 1.53 mm, followed by the methanol (23 ± 3.51 mm) and cold aqueous extract (22 ± 2.65 mm) against a NARST strain (Figure 2, X3). Even though there were differences in the ZOIs recorded for the methanol and the hot and cold aqueous extracts of both *A. africana* and *M*. *esculenta,* there was no significant difference in the zones of inhibition of these different solvent extracts against the test organisms at *p* <  0.05.

The slight difference in activity between the hot and cold aqueous and methanol extracts could be attributed to the differences in the compounds identified through GC-MS since the preliminary phytochemical screening revealed similar bioactive constituents in the various plant extracts. Though the compounds identified have other pharmacological activities, a few of them have been reported to exhibit antibacterial activity. Lupeol á-amyrin found in the ethyl acetate extract of *M. esculenta* is reported to exhibit antibacterial activity [31]. Reports indicate that D-mannose which was identified in the cold aqueous extract of *M*. *esculenta* can inhibit bacterial adhesion to the urothelium after oral intake [32]. Therefore, the anti-*Salmonella* activity exhibited by the extracts of *M*. *esculenta* and *A*. *africana* could be attributed to the presence of some of these compounds.

The *M*. *esculenta* cold aqueous extract at 100 mg/mL had the lowest ZOI of 18.0 ± 1.53 mm. However, Adam et al. [33] reported a ZOI of 17 mm and no inhibition with aqueous extract of bulb and peels, respectively. The findings of Adam et al. had lower anti-*Salmonella* activities compared to this current study (18.0 ± 1.53 mm), which can be attributed to the differences in the plant part used.

The ethyl acetate extracts of *A. africana* and *M*. *esculenta* had the least zones of inhibitions and better minimum inhibitory concentration (MIC) and minimum bactericidal concentration (MBC) values, and the petroleum ether extracts of both plants showed no activity against the test organisms, both in the agar well diffusion experiments and the broth dilution assay, compared to the aqueous and methanol extracts. The superior anti-*Salmonella* activity of the aqueous and methanol extracts justifies the principle observed in herbal practitioners' preference for using local gin as an extraction agent [34], and sometimes water.

The FTIR analysis of the various plant extracts showed the presence of functional groups such as S=O bending, C-N stretching, O-H bending, C=C bending, O-H stretch, C-O stretching, C-H stretching, N-H stretching, and C-H and C=O stretch (Table 4). The presence of these functional groups is indicative of compounds such as aldehydes, phenols, flavonoids, amines, and ketones, [35], of which flavonoids have been reported to exhibit antibacterial activities [36].

The presence of phytochemical compounds in *M*. *esculenta* and *A. africana* might have contributed to their anti-*Salmonella* activity since it is known that the active compounds produced by plants inhibit the life process of microbes especially the disease-causing ones [36]. For instance, tannins, alkaloids, flavonoids, and saponins glycosides have been reported to exhibit antibacterial activity [37]. Again, preliminary phytochemical screening showed the presence of saponins, glycosides, tannins and flavonoids in all the polar solvent extracts but not the nonpolar, which could also contribute to the better anti-*Salmonella* activity exhibited by the polar extracts for both *M*. *esculenta* and *A. africana*, most especially the aqueous extracts.

## 5. Conclusion

Hot water extracts of *A*. *africana* and *M*. *esculenta* had the highest percentage yield. Phytochemical screening revealed the presence of tannins, alkaloids, glycosides, saponins, and flavonoids in the plant extracts. Cold aqueous, hot aqueous, and methanol extracts of both plants had good anti-*Salmonella* activity, while ethyl acetate extracts had a weak anti-*Salmonella* activity. Petroleum ether extracts had no inhibitory effect against the test microorganisms. Hot aqueous extracts of *M*. *esculenta* and cold and hot aqueous extracts of *A. africana* had the best bactericidal effect on the MDRST. The methanol and aqueous (hot and cold) extracts of both *A*. *africana* and *M*. *esculenta* showed the best bactericidal effect against the SS. Methanol extract of *A*. *africana* had the best inhibitory effect against CRST. Aqueous extract of *A*. *africana* had the best inhibitory effect on the NARST. FTIR and GC-MS analysis showed the presence of probable compounds, some with reported antibacterial activity. These findings confirm the traditional usage of the plants and provide a scientific basis for their use in traditional medicines in the treatment of typhoid fever.

## Figures and Tables

**Table 1:** Percentage yield of crude extracts of *M. esculenta* and *A. africana*.

Solvents	Percentage yield (%)	
	*Aspilia africana*	*Manihot esculenta*
Aqueous (cold)	25.42	32.28
Aqueous (hot)	31.02	35.63
Methanol	20.95	24.78
Ethyl acetate	13.68	14.64
Petroleum ether	10.25	12.30

**Table 2:** Phytochemical composition of *M. esculenta* and *A. africana* extracts.

Plant	Secondary metabolites	Pulverized sample	P_ET_	E_TA_	M_ET_	A_QC_	A_QH_
*M. esculenta*	Tannins	+	−	−	+	+	+
	Saponins	+	−	−	+	+	+
	Glycosides	+	−	−	+	+	+
	Alkaloids	+	+	+	+	+	+
	Flavonoids	+	−	−	+	+	+
	Sterols	−	−	−	−	−	−
	Triterpenoids	+	+	+	+	−	−

*A. africana*	Tannins	+	−	−	+	+	+
	Saponins	+	−	−	+	+	+
	Glycosides	+	−	−	+	+	+
	Alkaloids	+	+	+	+	+	+
	Flavonoids	+	−	−	+	+	+
	Sterols	−	−	−	−	−	−
	Triterpenoids	+	+	+	+	−	−

+, phytoconstituent is present; −, phytoconstituent is absent; P_ET_, petroleum ether extract; E_TA_, ethyl acetate extract; M_ET_, methanol extract; A_QC_, cold aqueous; A_QH_, hot aqueous.

**Table 3:** IR spectra of functional groups present in various extracts of *Aspilia africana*.

Crude extract	Peak value (cm^−1^)	Functional group
Methanol extract	1032.84	S=O stretching
	1241.18	C-N stretching
	1450.73	O-H bending
	1707.72	(C=C) stretching
	2852.88	Csp3-H stretching
	3388.46	O-H stretch

Aqueous extract	1043.46	S=O stretching
	1258.25	C-O stretching
	1390.17	C-H stretching
	1580.91	N-H bending/C=C stretch
	2919.66	Csp3-H stretching
	3255.02	O-H stretch

Ethyl acetate extract	898.72	C-O stretch
	1089.68	C=C bending
	1166.35–1243.19	C-N stretch
	1376.39	O-H bending
	1451.25	C-H bending
	1732.38	C=O stretch
	2852.83	Csp3-H stretching

Petroleum ether	970.37–1093.40	C=C bending/C-O stretch
	1732.34–1243.84	C-N stretch
	1376.28	O-H bending
	1451.50	C-H bending
	1732.56	C=O stretch
	2851.22–2922.37	Csp3-H stretch

**Table 4:** IR spectra of functional groups present in various extracts of *Manihot esculenta*.

Crude extract	Peak value (cm^−1^)	Functional group
Methanol extract	1041.69	CO-O-CO/S=O stretch
	1262.81	S=O/C-O/C-N stretch
	1409.17	C=C stretch
	1587.97	Csp3-H
	2917.07	O-H stretch

Aqueous extract	3234.28	CO-O-CO/S=O stretch
	1262.81	S=O/C-O/C-N stretch
	1409.17	C=C stretch
	1587.97	Csp3-H
	2917.07	O-H stretch
	3234.28	CO-O-CO/S=O stretch

Ethyl acetate extract	1036.17	S=O stretch
	1164.12	C-O stretch
	1457.53	O-H bending
	1498.70	C-H stretch
	1736.57	C=O stretch
	2852.31–2922.38	Csp3-H stretch
	3389.59	O-H stretch

Petroleum ether	972.89	S=O stretch
	1084.45	C=C bending
	1166.78	C-O stretch
	1376.70–1454.77	C-H bending
	1701.15	C=O stretch
	2851.72–2921.71	Csp3-H

**Table 5:** MICs of extracts of *A. africana* and *M. esculenta* against test organisms.

Minimum inhibition concentration (mg/mL)							
Plants	Organism codes	M_ET_	A_QC_	A_QH_	E_TA_	P_ET_	C_IP_
*Multidrug-resistant S. typhi*							
*A. africana*	G10163	5.0	5.0	2.5	N/I	N/I	1 × 10^−2^
	G10209	40.0	2.5	2.5	40.0	N/I	1 × 10^−2^
	G10325	5.0	10.0	2.5	10.0	N/I	1 × 10^−2^
	G10194	20.0	10.0	10.0	N/I	N/I	1 × 10^−3^
	G10573	10.0	5.0	10.0	40.0	N/I	3 × 10^−3^
	(SS)	5.0	10.0	5.0	20.0	N/I	1 × 10^−3^
*M. esculenta*	G10163	20.0	5.0	2.5	40.0	N/I	1 × 10^−2^
	G10209	40.0	5.0	2.5	N/I	N/I	1 × 10^−2^
	G10325	20.0	5.0	10.0	20.0	N/I	1 × 10^−2^
	G10194	10.0	20.0	20.0	20.0	N/I	1 × 10^−3^
	G10573	20.0	40.0	40.0	20.0	N/I	3 × 10^−3^
	(SS)	10.0	20.0	5.0	20.0	N/I	1 × 10^−3^

*Ciprofloxacin-resistant S. typhi*							
*A. africana*	G10628	10.0	5.0	5.0	N/I	N/I	1 × 10^−2^
	G20247	5.0	5.0	5.0	40.0	N/I	1 × 10^−2^
	G11082	20.0	5.0	10.0	40.0	N/I	1 × 10^−2^
	G10692	10.0	10.0	10.0	40.0	N/I	1 × 10^−2^
	G10713	5.0	10.0	5.0	N/I	N/I	1 × 10^−2^
	(SS)	5.0	10.0	5.0	20.0	N/I	1 × 10^−3^
*M. esculenta*	G10628	20.0	20.0	40.0	N/I	N/I	1 × 10^−2^
	G20247	5.0	5.0	40.0	40.0	N/I	1 × 10^−2^
	G11082	40.0	10.0	40.0	40.0	N/I	1 × 10^−2^
	G10692	20.0	20.0	40.0	40.0	N/I	1 × 10^−2^
	G10713	20.0	10.0	40.0	N/I	N/I	1 × 10^−2^
	(SS)	5.0	10.0	10.0	20.0	N/I	1 × 10^−3^

*Nalidixic acid-resistant S. typhi*							
*A. africana*	G10037	10.0	5.0	10.0	40.0	N/I	1 × 10^−3^
	G20157	10.0	5.0	10.0	40.0	N/I	1 × 10^−2^
	G20014	5.0	10.0	5.0	N/I	N/I	3 × 10^−3^
	(SS)	5.0	10.0	5.0	20.0	N/I	1 × 10^−3^
*M. esculenta*	G10037	40.0	20.0	40.0	N/I	N/I	1 × 10^−3^
	G20157	5.0	20.0	20.0	N/I	N/I	1 × 10^−2^
	G20014	10.0	20.0	40.0	20.0	N/I	3 × 10^−3^
	(SS)	10.0	20.0	10.0	20.0	N/I	1 × 10^−3^

N/I, no inhibition; P_ET_, petroleum ether extract; E_TA_, ethyl acetate extract; M_ET_, methanol extract; A_QC_, cold aqueous; A_QH_, hot aqueous; C_IP_, ciprofloxacin; SS, sensitive strain.

**Table 6:** MBC of extracts of *A. africana* and *M. esculenta* against test organisms.

Minimum bactericidal concentration (mg/mL)							
Plants	Organism codes	M_ET_	A_QC_	A_QH_	E_TA_	P_ET_	C_IP_
*Multidrug-resistant S. typhi*							
*A. africana*	G10163	10.0	10.0	5.0	N/I	N/I	1 × 10^−2^
	G10209	80.0	5.0	5.0	80.0	N/I	1 × 10^−2^
	G10325	10.0	20.0	5.0	20.0	N/I	1 × 10^−2^
	G10194	40.0	20.0	20.0	N/I	N/I	1 × 10^−2^
	G10573	20.0	10.0	20.0	80.0	N/I	1 × 10^−2^
	(SS)	10.0	20.0	10.0	40.0	N/I	1 × 10^−3^
*M. esculenta*	G10163	40.0	10.0	5.0	80.0	N/I	1 × 10^−2^
	G10209	80.0	10.0	5.0	N/I	N/I	1 × 10^−2^
	G10325	40.0	10.0	20.0	40.0	N/I	1 × 10^−2^
	G10194	20.0	40.0	40.0	40.0	N/I	1 × 10^−2^
	G10573	40.0	80.0	80.0	40.0	N/I	1 × 10^−2^
	(SS)	20.0	40.0	10.0	40.0	N/I	1 × 10^−3^

*Ciprofloxacin-resistant S. typhi*							
*A. africana*	G10628	20.0	10.0	10.0	N/I	N/I	N/I
	G20247	10.0	10.0	10.0	80.0	N/I	N/I
	G11082	40.0	10.0	20.0	80.0	N/I	N/I
	G10692	20.0	20.0	20.0	80.0	N/I	N/I
	G10713	10.0	20.0	10.0	N/I	N/I	N/I
	(SS)	10.0	20.0	10.0	40.0	N/I	1 × 10^−3^
*M. esculenta*	G10628	40.0	40.0	80.0	N/I	N/I	N/I
	G20247	10.0	10.0	80.0	80.0	N/I	N/I
	G11082	80.0	20.0	80.0	80.0	N/I	N/I
	G10692	40.0	40.0	80.0	80.0	N/I	N/I
	G10713	40.0	20.0	80.0	N/I	N/I	N/I
	(SS)	10.0	20.0	20.0	40.0	N/I	1 × 10^−3^

*Nalidixic acid-resistant S. typhi*							
*A. africana*	G10037	20.0	10.0	20.0	80.0	N/I	1 × 10^−3^
	G20157	20.0	10.0	20.0	80.0	N/I	1 × 10^−2^
	G20014	10.0	20.0	10.0	N/I	N/I	3 × 10^−3^
	(SS)	10.0	20.0	10.0	40.0	N/I	1 × 10^−3^
*M. esculenta*	G10037	80.0	40.0	80.0	N/I	N/I	1 × 10^−3^
	G20157	10.0	40.0	40.0	N/I	N/I	1 × 10^−2^
	G20014	20.0	40.0	80.0	40.0	N/I	3 × 10^−3^
	(SS)	20.0	40.0	20.0	40.0	N/I	1 × 10^−3^

N/I, no inhibition; P_ET,_ petroleum ether extract; E_TA_, ethyl acetate extract; M_ET_, methanol extract; A_QC,_ cold aqueous; A_QH_, hot aqueous; C_IP_, ciprofloxacin; SS, sensitive strain.

**Table 7:** Compounds identified in *A. africana* by GC-MS analysis.

Peak number	Retention time (min)	Compound name	Molecular formula	Molecular weight (g/mol)	Peak area (%)
*Methanol extract*					
1	8.30	1-Heptatriacontanol	C_17_H_36_O	256.5	2.72
2	10.96	9-Octadedecenoic acid	C_18_H_34_O_2_	282.5	1.64
3	11.47	4-((IE)-3-hydroxy-1-propenyl)-2-methoxyphenol	C_10_H_12_O_3_	180.2	2.59
4	12.51	2-Pendadecanone	C_15_H_30_O	226.4	1.26
5	13.89	*n*-Hexadecanoic acid	C_16_H_32_O_2_	256.4	19.29
6	14.35	Benzoic acid	C_6_H_5_COOH	122.1	3.28
7	14.46	2,4,6-Decatrienoic acid	C_10_H_14_O_2_	166.2	2.61
8	15.79	Phtyol	C_20_H_40_O	296.53	5.71
9	16.24	9,12,15-Octadecatrienoic acid (Z, Z, Z)	C_18_H_30_O_2_	278.4	9.55
10	18.09	Cyclopentolate	C_17_H_25_NO_3_	291.39	1.89
11	19.21	9-Octadecenamide (Z)	C_18_H_35_NO	281.48	2.78
12	20.07	á–Pimaric acid	C_20_H_30_O_2_	302.46	5.20
13	20.60	2H-benzo [f]oxirenol[2,3-E]benzofuran-8(9H)-one	C_19_H_32_N_2_O_3_	336.5	0.97

*Cold aqueous extract*					
1	29.49	Octasiloxane 1,1,3,3,5,5,7,7,9,9,11,11,13,13,15,15-hexadecamethyl	C_18_H_54_O_7_Si_3_	607.3	1.46
2	30.58	18-Pentatriacontanone	C_35_H_70_O	506.9	1.71
3	32.19	Cyclotrisiloxane hexamethyl	C_6_H_18_O_3_Si_3_	222.46	1.19
4	33.99	2,4-Dihydroxyacetophenone, bis(trimethylsilyl)ether	C_14_H_24_O_3_Si_2_	296.51	1.31
5	35.47	Phosphorothioic acid	H_3_PO_3_S	114.06	29.71

*Hot aqueous extract*					
1	17.31	Cis-11-eicosenamide	C_20_H_39_NO	309.5	5.05
2	18.59	Hexadecanoic acid	C_16_H_32_O_2_	256.4	2.90

*Ethyl acetate extract*					
1	15.78	Phytol	C_20_H_40_O	296.53	0.29
2	16.16	Z-(13,14-Epoxy)tetradec-11-en-1-ol-acetate	C_16_H_28_O_3_	268.39	0.19
3	20.03	Abietic acid á-Pimaric acid	C_20_H_30_O_2_	302.45	0.62
4	22.10	1-Monolinoleoylglycerol trimethylsilyl ether	C_27_H_56_O_4_Si_2_	500.9	0.24
5	24.30	Octasiloxane 1,1,3,3,5,5,7,7,9,9,11,11,13,13,15,15-hexadecamethyl	C_18_H_54_O_7_Si_3_	607.3	0.16
6	25.20	Octasiloxane 1,1,3,3,5,5,7,7,9,9,11,11-dodecamethyl	C_16_H_48_O_7_Si_8_	577.2	0.85
7	26.01	Urs-12-en-28-oic acid 3-hydroxy-methylester	C_31_H_50_O_3_	470.38	2.04
8	29.27	Hexasiloxane 1,1,3,3,5,5,7,7,9,9,11,11-dodecamethyl	C_12_H_36_O_5_Si_6_	428.92	22.11
9	31.99	Stigmasterol	C_29_H_48_O	412.69	18.70
10	33.18	ç-Sitosterol á-Sitosterol	C_29_H_50_O	414.7	1.55
11	33.84	á-Amyrin	C_30_H_50_O	426.7	4.27
12	34.30	4,4,6a,6b,8a,11,12,14b-Octamethyl-	C_32_H_50_O_2_	466.7	2.43
13	34.88	á-Amyrin	C_30_H_50_O	426.7	6.65
14	36.44	12-Oleanen-3-yl acetate	C_32_H_52_O_2_	468.8	9.13
15	36.63	Glycocholic acid	C_26_H_43_NO_6_		0.55
16	37.63	12-Oleanen-3-yl acetate	C_32_H_52_O_2_	468.8	17.11

**Table 8:** Compounds identified in *M. esculenta* by GC-MS analysis.

Peak number	Retention time (min)	Compound name	Molecular formula	Molecular weight (g/mol)	Peak area (%)
*Methanol extract*					
1	13.84	*n*-Hexadecanoic acid	C_16_H_32_O_2_	256.4	8.69
2	15.78	Phytol	C_20_H_40_O	296.53	5.45
3	16.15	9,12,15-Octadecatrienoic acid (Z, Z, Z)	C_18_H_30_O_2_	278.43	12.78

*Cold aqueous extract*					
1	4.15	4H-Pyran-4-one, 2,3-dihydro-3,5-dihydroxy-6-methyl	C_6_H_8_O_4_	144.12	2.79
2	5.03	Benzofuran,2,3-dihydro	C_8_H_8_O	120.15	3.80
3	6.34	Benzaldehyde, 2-methyl	C_8_H_8_O	120.15	4.76
4	8.00	2-Methyl-4-vinylphenol	C_9_H_10_O	134.17	1.78
5	10.50	(3-Nitrophenyl)methanol acetate (ester)	C_8_H_7_NO_4_	181.15	6.51
6	13.67	Dasycarpidan-1-methanol, acetate (ester)	C_20_H_26_N_2_O_2_	326.4	1.51
7	14.79	D-Mannose	C_6_H_12_O_6_	180.16	11.84
8	16.44	10,12-Docosadiynedioic acid ditms	C_2_H_50_O_4_Si_2_	506.9	2.92
9	19.19	9-Octadecenamide, (Z)	C_18_H_35_NO	281.48	1.11

*Hot aqueous extract*					
1	4.40	4H-Pyran-4-one, 2,3-dihydro-3,5-dihydroxy-6-methyl	C_6_H_8_O_4_	144.12	1.61
2	5.23	Benzofuran,2,3-dihydro	C_8_H_8_O	120.15	1.99
3	6.46	2-Methoxy-4-vinylphenol	C_9_H_10_O	134.17	1.18
4	11.13	2-Cyclohexen-1-one, 4-(3-hydroxybutyl)-3,5,5-trimethyl	C_13_H_22_O_2_	210.31	1.48
5	12.07	5,5,8a-Trimethyl-3,5,6,7,8,8a-hexahydro-2H-chromene	C_12_H_20_O	180.29	1.29
6	13.66	*n*-Hexadecanoic acid	C_16_H_32_O_2_	256.4	1.34
7	15.53	Octadecanoic acid	C_18_H_36_O_2_	284.48	37.59
8	17.29	9-Octadecenamide	C_18_H_35_NO	281.48	9.59
9	18.59	Hexadecanoic acid, 2-hydroxy-1-(hydroxymethyl) ethyl ester	C_19_H_38_O_4_	330.50	4.74

*Ethyl acetate extract*					
1	15.78	Phytol	C_20_H_40_O	296.53	0.06
2	19.19	9-Octadecenamide	C_18_H_35_NO	281.48	0.03
3	31.53	Octasiloxane 1,1,3,3,5,5,7,7,9,9,11,11,13,13,15,15-hexadecamethyl	C_16_H_48_O_7_Si_8_	577.2	42.46
4	31.99	C(14a)–homo-27-norgammacer-14-ene	C_31_H_52_O_2_	456.7	6.29
5	32.86	D-Friedoolean-14-en-3-one	C_30_H_48_O	424.7	6.18
6	33.16	Betulin			
Lupeol	C_30_H_50_O_2_	442.72	7.03		
7	33.45	6a,14a-Methanopicene,perhydro-1,2,4,6b,9,9,12a-heptamethyl-10-hydroxy	C_30_H_50_O	426.7	8.24
8	33.86	á-Amyrin	C_30_H_50_O	426.7	7.16
9	34.39	Lup-20(29)-en-3-one	C_30_H_48_O	424.7	6.84
10	34.92	Lupeol á-amyrin	C_30_H_50_O_2_	442.72	4.06
11	35.54	17.Alfa.21á-28, 30-bisnorhopane	C_28_H_48_	384.7	1.76
12	36.28	6a,14a-Methanopicene,perhydro-1,2,4a,6b,9,9,12a-heptamethyl-10-hydroxy	C_30_H_50_O	426.7	2.13

## Data Availability

The dataset obtained is included within the article.
